# Supplementary material for: APC mutations in human colon lead to decreased neuroendocrine maturation of ALDH+ stem cells that alters GLP-2 and SST feedback signaling: Clue to a link between WNT and retinoic acid signalling in colon cancer development
Source: PLoS One. 2020 Oct 28;15(10):e0239601. doi: 10.1371/journal.pone.0239601 (PMC7592776; doi:10.1371/journal.pone.0239601)

Supplemental Materials

Mathematical model of neuroendocrine cell dynamics in normal and neoplastic colon.

Our goal was to create a model based on ordinary differential equations that simulates maturation of stem cells along the neuroendocrine lineage. We constructed the model in order to understand the mechanism underlying the different steady states that arise in colon tissue due to *APC* mutations (normal, FAP, adenoma and CRC).

Model Design - the following methods and approaches were used in our modeling

We constructed our model based on a modified version of a simple 3 compartment nonlinear model described by Nakata et al [32]. Our model is based on the assumption that the differentiation of cells takes place during their division. In the model mechanism, a stem cell divides and gives rise to two daughter cells: each of which can be a stem cell or a progenitor cell. Similarly, when a progenitor cell divides, it can give rise to progenitor cells or mature cells. Cell division is described by a division rate and a probability of self-renewal. The rate of cell division is regulated by a feedback loop from the mature NE cell compartment. Because the probabilities for self-renewal and maturation must add to 100%, the probability of self-renewal thus provides a measure of the probability of maturation (into progenitor or mature cells).

Model Parameters.

Let $w(t)$, $u(t)$, and $v(t)$ denote the concentrations of stem, progenitor, and mature cells, respectively, as a function of time, t. Let a_w and a_u be the probability of self-renewal of stem cells and progenitor cells, respectively, whereby a_w, $a_u \in [0,1)$. Let the terms d_w and d_u denote the rates of cell division – rates that are regulated by the number of mature cells. That is, when there are enough mature cells, the ability of the stem cells and progenitor cells to self renew or mature is adjusted to compensate for the loss of colonic crypt cells due to cell death. This simulates the feedback loop involving regulation by the mature NE cells. Therefore, the rates of cell division are given by

$$d_w(v) = \frac{p_w}{1+kv}, \qquad d_u(v) = \frac{p_u}{1+kv},$$

where p_w and p_u are positive constants and k is a positive constant that measures the sensitivity to the influence of v. We can see here, that as v becomes large the division rates of stem and progenitor cells tends to zero. These functions provide the feedback mechanism that describes the regulatory behavior exerted by the mature NE cells. Finally, let the constants μ_w, μ_u, and μ_v be the rates of death of each cell type.

A schematic of the reaction kinetics are given in the following figure.

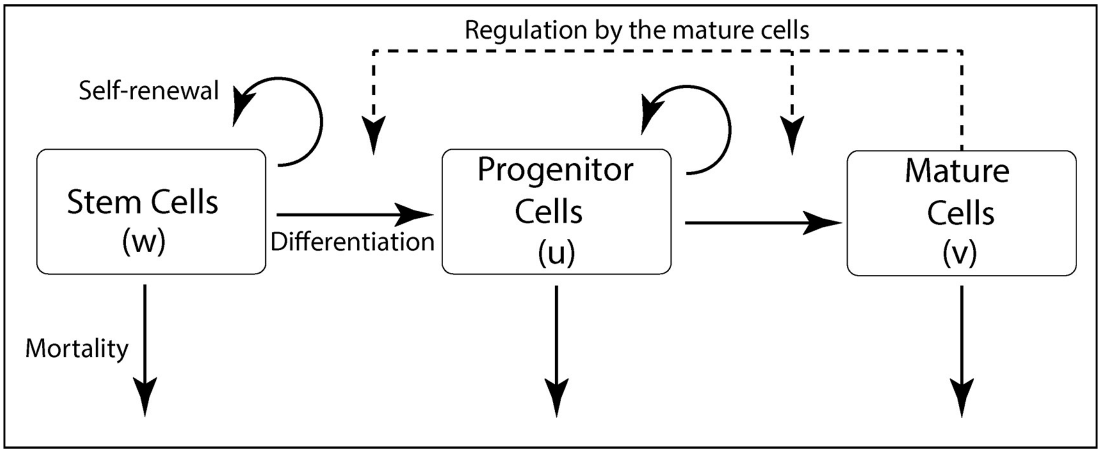

We can describe the inflow and outflow of stem cells from or to the different compartments as follows. Consider the possibility of a stem cell dividing symmetrically into two stem cells. Then the probability of renewal is exactly $a_w = 1$ and the net production of stem cells is exactly one new stem cell. However, if the stem cell divides asymmetrically then the renewal probability is $a_w = 0$ and the stem cell compartment loses one cell. Therefore, for any probability a_w, the net flux in the stem cell compartment due to cell division is given by $(2a_w - 1)$. Now, to describe the overall change in the stem cell compartment with time, we have to multiply this number by the division rate $d_w(v)$. Incorporating the death rate of stem cells , μ_w, the change in the stem cell compartment is given by

$$\frac{dw}{dt} = (2a_w - 1)d_w(v)w - \mu_w w.$$

Similarly, the change in the progenitor cell compartment due to division of progenitor cells is $(2a_u - 1)\, d_u(v)u$. Furthermore, the inflow from the stem cell compartment into the progenitor cell compartment is given by $2(1-a_w)\, d_w(v)w$. Combining these terms with the death rate of progenitor cells, μ_u, the change in the progenitor cell compartment is

$$\frac{du}{dt} = (2a_u - 1)d_u(v)u + 2(1 - a_w)d_w(v)w - \mu_u u$$

Finally, the inflow to the mature cell compartment from the progenitor cell compartment, is described by $2(1-a_u)\, d_u(v)u$. Combined with the death rate of mature cells, the change in the mature cell compartment is:

$$\frac{dv}{dt} = 2(1 - a_u)d_u(v)u - \mu_v v.$$

The system is non-dimensionalized in the following way. The time variable is scaled by the death rate of stem cells, i.e. $\tau = \mu_w t$. The actual cell populations are not scaled, but we do define the following scaled parameters

$$r_w = \frac{p_w}{\mu_w}, \qquad r_u = \frac{p_u}{\mu_w}, \qquad m_u = \frac{\mu_u}{\mu_w}, \qquad m_v = \frac{\mu_v}{\mu_w}.$$

This gives the following nondimensional system:

$$\begin{aligned}
\frac{dw}{d\tau} &= \frac{(2a_w - 1)r_w}{1 + kv} w - w \\
\frac{du}{d\tau} &= \frac{(2a_u - 1)r_u}{1 + kv} u + \frac{2(1 - a_w)r_w}{1 + kv} w - m_u u \\
\frac{dv}{d\tau} &= \frac{2(1 - a_u)r_u}{1 + kv} u - m_v v
\end{aligned}$$

The reproduction number, which is defined as the number of cells coming in via self-renewal minus the number of cells going out via cell division, for stem cell and proliferating cell compartments is given by:

$$R_w = (2a_w - 1)r_w, \qquad R_u = \frac{(2a_u - 1)r_u}{m_u}.$$

According to the stability analysis provided by Nakata et al [32], a positive equilibrium solution exists if $R_w > 1$ and $R_w > R_u$. This fits with the fact that at equilibrium progenitor cells should self-renew less frequently than stem cells, as the former have additional inflow from the latter. Furthermore, the instability value decreases with the increasing rate of death of mature cells, m_v.

The nontrivial positive expressions for stem cells, progenitor cells and mature cells, w^*, u^*, and v^* at steady state are given by

$$w^* = \frac{m_u(R_w - R_u)}{2(1 - a_w)r_w} u^*$$

$$u^* = \frac{m_v R_w}{2(1 - a_u)r_u} v^*$$

$$v^* = \frac{1}{k}(R_w - 1).$$

$$\frac{w^*}{u^*} = \frac{m_u(R_w - R_u)}{2(1 - a_w)r_w}, \qquad \frac{u^*}{v^*} = \frac{m_v R_w}{2(1 - a_u)r_u}.$$

Iterative fitting to find model parameter values that correspond to normal and abnormal steady state proportions in the crypt.

Nontrivial steady state solutions were used to find a set of fitted parameters for each type of colonic tissue. Given the steady state expressions above, and the ratios SC/PC and PC/MC (based on biologic data), we determined a set of model parameters that fit with each of the ratios (w^*/u^*, u^*/v^*) of each pathophysiologic state. The parameters a_u, r_u, and m_u were fixed (kept constant). The parameter k was set to equal 1 because it does not influence the ratio; it only influences the number of cells in the steady state. Table 1 gives percentages of cell types for normal crypts, FAP crypts, adenomas, and colon cancers based on various staining results (see Figure 2). It also shows the results of iterative fitting of model parameters to the biologic data. There was an excellent fit: the relative error for the steady state percentages compared to the actual biological percentages were 0.006 for normal, 0.005 for FAP, 0.001 for adenoma, and 0.001 for CRC.

Table 1. Comparison of (i) Proportions of cell types and (ii) model fit, for normal (wild type) and mutant (neoplastic) colonic tissues.

	% SC	w^* %	%PC	u^* %	%MC	v^*%	SC/PC	w^*/u^*	PC/MC	u^*/v^*
Normal	18.95	18.62	76.84	77.14	4.21	4.24	0.25	0.2415	18.2	18.20
FAP	22.22	21.94	51.11	51.14	26.67	26.92	0.43	0.4290	1.9	1.900
Adenoma	71.84	71.80	19.42	19.39	8.74	8.81	3.7	3.7039	2.2	2.199
CRC	80.95	81.01	12.70	12.66	6.35	6.33	6.4	6.4002	2.0	2.000

%SC is the percent of cells that stain only for ALDH1. %PC is the percent of cells that co-stain for ALDH1 and CGA. %MC is the percent of cells that stain only for CGA (see bar graph in Fig 3).

We then determined how the parameters a_w and r_w changed, because these values represent changes in stem cell behavior (number of SCs or increased stemness) in the different tissue types that was due to *APC* mutations. Furthermore, we determined changes in the parameter m_v, which denotes the death rate of mature cells. This value may be influenced by an abnormal APC gradient in the crypt. We assumed that $a_w > a_u$ and $r_w < r_u$ because stem cells are more likely to self-renew, but they divide slower. In addition, we assumed for the death rates that $m_v > m_u > 1$ since both progenitor cells and mature cells die faster than stem cells. Finally, we set parameters a_u, r_u, and m_u such that the reproduction number of progenitor cells, R_u, equals 1. Table 2 below shows the parameter values determined from our modeling after iterative fitting as discussed above.

Table 2: Parameter fit with corresponding cell type densities.

Parameter*	Normal	*FAP*	Adenoma	Cancer
a_u	0.55	0.55	0.55	0.55
a_w	0.600	0.639	0.871	0.918
r_u	15	15	15	15
r_w	14.04	13.98	9.53	7.34
m_u	1.5	1.5	1.5	1.5
m_v	87.5	6.6	4.2	4.4
R_u	1	1	1	1
R_w	2.8080	3.8864	7.0713	6.1362
k	1	1	1	1
Ratios	Normal	*FAP*	Adenoma	Cancer
$w^*\%$	18.62	21.94	71.80	81.01
$u^*\%$	77.14	51.14	19.39	12.66
$v^*\%$	4.24	26.92	8.81	6.33
w^*/u^*	0.2415	0.4290	3.7039	6.4002
u^*/v^*	18.2000	1.9000	2.1999	2.0000
Error†	0.006	0.005	0.001	0.001

* a_u and a_w represent the renewal probability of progenitor cells and stem cells, respectively. r_u and r_w denote the division rates of progenitor cells and stem cells, respectively. m_u and m_v are the death rates of progenitor cells and mature cells, respectively. R_u and R_w (defined above) denote the reproduction numbers of the progenitor and stem cells, respectively. k is the parameter that controls sensitivity of the feedback loop.
† This is the relative error for the determined steady state percentages compared to the actual biological percentages given in Table 1.

We notice here that the death rate for mature cells in a normal crypt, m_v = 87.5, stands out among the rest. This is due to the high progenitor cell count and very low mature cell count for normal crypts. This does make sense, however, in order to achieve the high ratio of u/v = 18.2. We can also see a significant increase in the renewal probability for stem cells, a_w, as the crypt status worsens. In cancerous crypts, the probability that a stem cell will self-renew is 91.8% compared to 60% in a normal crypt. We also see a decline in the division rate of stem cells, r_w, as the crypt becomes cancerous. In a cancerous crypt, stem cells divide at a rate of 7.34 versus the rate of 14.04 in a normal crypt, which implies that stem cells in cancerous crypts divide almost half the speed as those in a normal crypt.

We also observe an increase in the stem cell reproduction number, R_w, in adenoma and cancer crypts. The normal stem cell reproduction number is 2.808 compared to 7.0713 and 6.1362 in adenoma and cancer, respectively. The last five rows of Table 2 confirm that the parameter choice matches the data well. The error computed in the last row is the relative error between the percentages of stem, progenitor, and mature cells in Table 1 and the determined percentages in Table 2. This particular parameter set yields a relative error on the order of 10^{-3} for each status of the crypt. The bar graphs in Figure 3 illustrate these results.

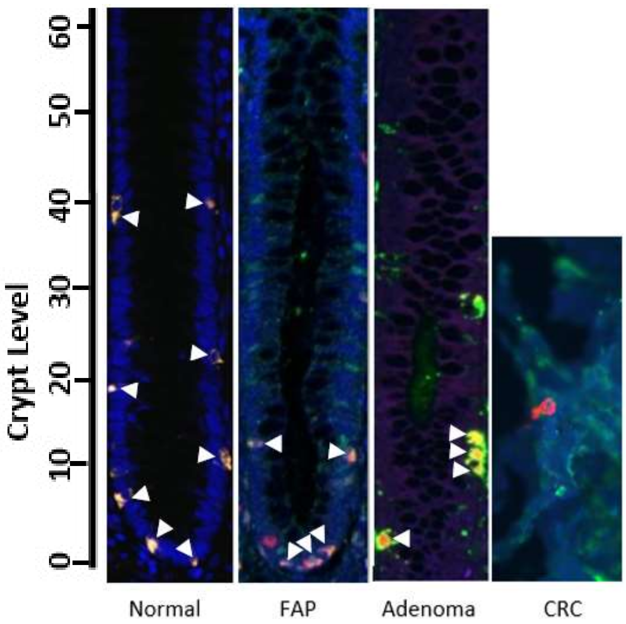

Supplemental Figure S1. Immunofluorescence co-staining analysis was done using human colonic tissues which have different *APC*-zygosity states for expression of ALDH1 (green) and CGA (red). Representative co-staining images are shown with arrowheads marking co-stained cells (yellow). Note. CRCs do not have crypt structures due to tissue disorganization.

Supplemental Figure S2. Raw flow cytometry data for plots shown in Figure 5.

Supplemental Figure S2A– ALDEFLUOR assay done on SW480 cells grown with and without exogenous SST

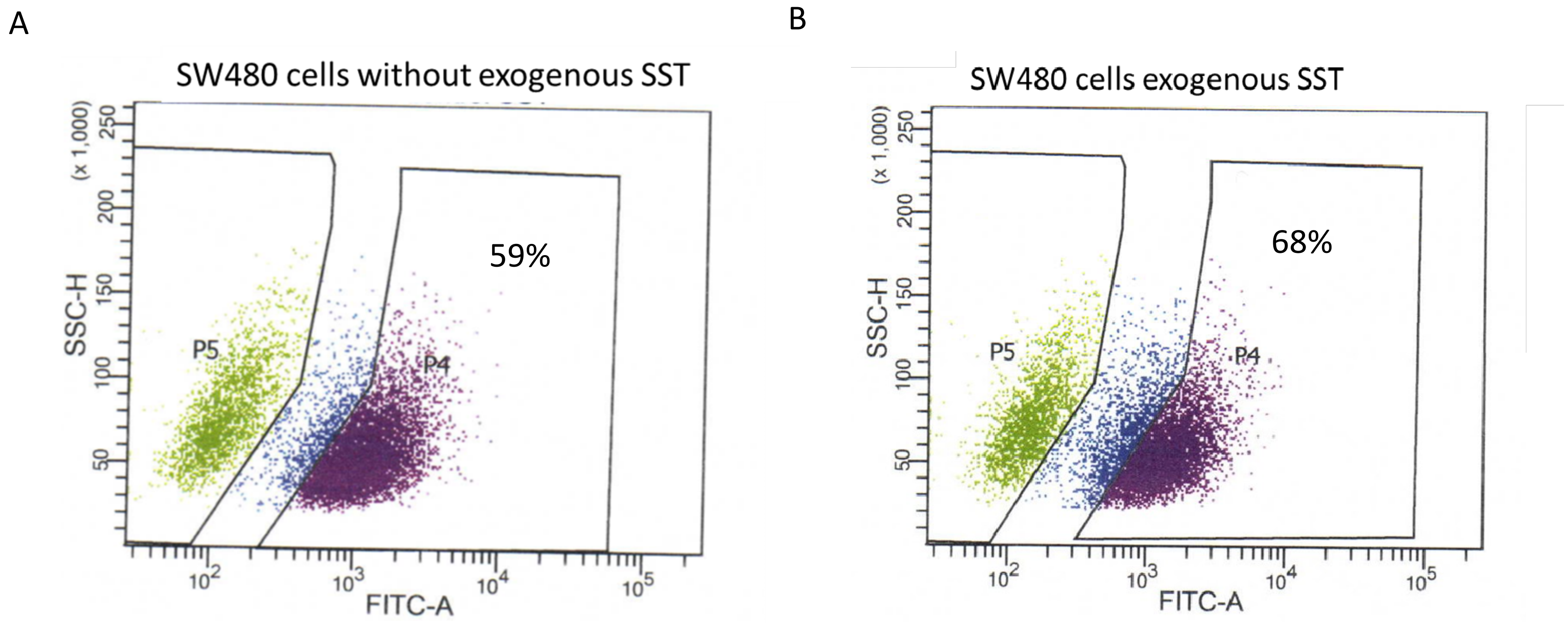

Supplemental Figure S2B– Aldefluor assay done on ALDH+ cells grown in either normal growth media or conditioned medium (CM) that was collected from SSSTR1+ cells growing in culture.

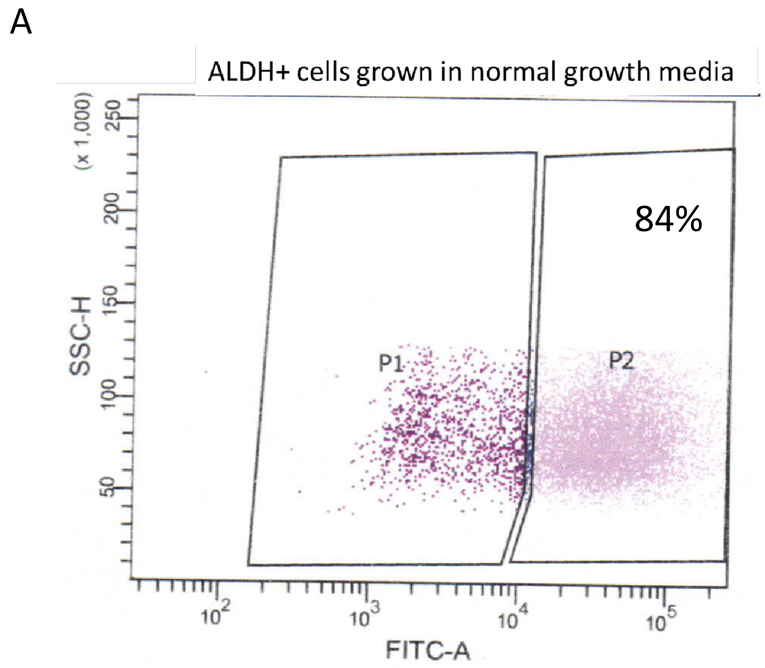

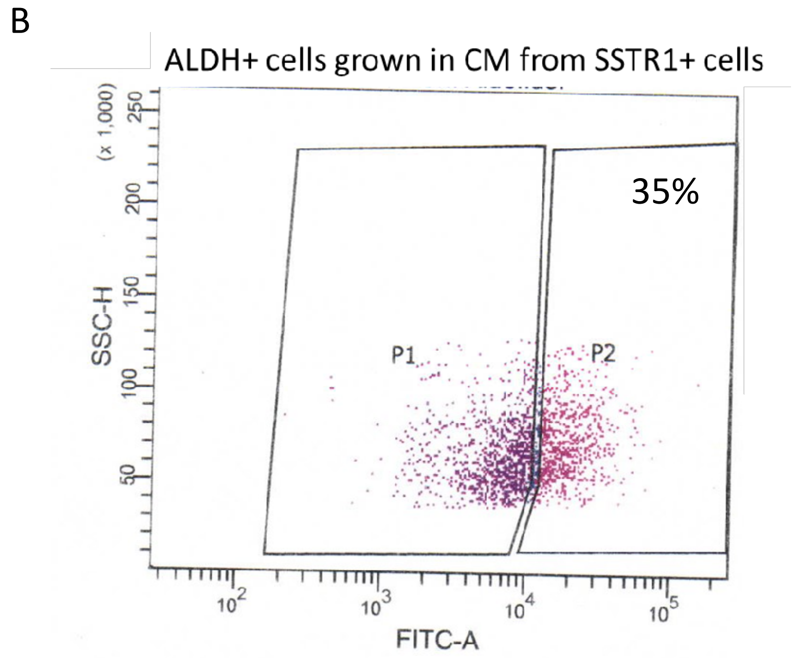

Supplemental Figure S2C– ALDEFLUOR assay done on co-culture of ALDH+, SSTR1+, and SSTR1- cell populations isolated from HT29 cells.

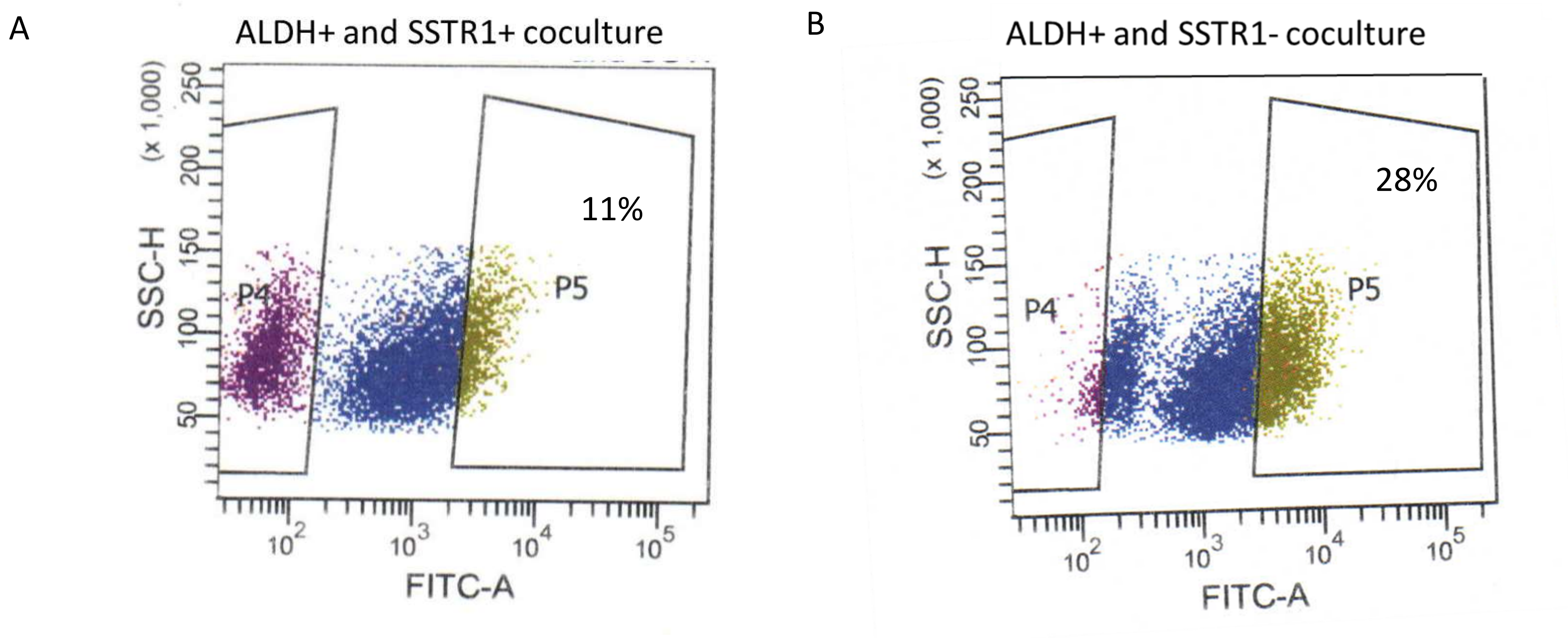

Supplement: S1 File — (PDF) [file pone.0239601.s001.pdf]
