# Supplementary material for: APC mutations in human colon lead to decreased neuroendocrine maturation of ALDH+ stem cells that alters GLP-2 and SST feedback signaling: Clue to a link between WNT and retinoic acid signalling in colon cancer development
Source: PLoS One. 2020 Oct 28;15(10):e0239601. doi: 10.1371/journal.pone.0239601 (PMC7592776; doi:10.1371/journal.pone.0239601)
Supplement: Attachment — Submitted filename: Responses to reviewers.docx [file pone.0239601.s002.docx]

We thank the reviewers for their helpful comments. Each reviewer comment (*italics*) is followed by our response (►).

**Reviewer #1:**

Major concerns:
1. The authors state, in the abstract and introduction, that they are investigating a correlation between Wnt signaling and RA signaling. The data shown in this manuscript have not proven this interaction.

►We have toned down our main conclusion so that it doesn’t imply that we discovered a correlation between Wnt signaling and RA signaling, but that the study provides a clue to a link between Wnt signaling and RA signaling.

2. The staining for ALDH1 and CGA in Figure 1 are described as having a similar pattern (1A and B – normal). However, the images shown do not depict this.

►We apologize. We have now revised the wording that refers to Figure 1 to indicate that the results based on staining indices (Figure 1C) shows that the crypt distribution of CGA+ cells and ALDH+ cells was similar.

3. The authors state that “the proportion of SSTR+ cells tended to be greater than that of GLP-2R+ cells” in the colon cancer cell lines. The data shown in Figure 5 depict that one of the cell lines (COLO320) has some differences between SSTR1 and GLP-2R, but the cell lines chosen for future studies (SW480 and HT29) show no difference in the percentage of positive cells for SSTR1 and GLP-2R. The in vitro studies are lacking error and significance designations (Figure 5B-H), making the data difficult to interpret. In addition, controls are lacking in Figure 5B. The authors state that GLP-2 decreases the ALDH+ cell population, but that SST does not. Without showing the control cells, it is impossible to discern the effect of SST and GLP-2.

►We thank the reviewer for pointing this out. The sentence stating “the proportion of SSTR+ cells tended to be greater than that of GLP-2R+ cells” is now deleted. We have now added error and significance designations for in vitro studies in Figures 5A-5D to make the data easier to interpret. We have clarified that Figures 5E-5H are representative results. In addition, we have now added controls in Figures 5B-5D to make it possible to discern the effect of SST, GLP-2, and conditioned medium.

4. The results describe data of CGA/ALDH double staining in FAP tissues, but FAP staining isn’t shown (Figure 2). It would be helpful to show the IF staining for normal, FAP, adenoma, and carcinoma, since those are shown in panel “F.” In addition, it would be helpful to show the crypt level for all four conditions.

► The purpose of the graphic in Figures 2A & 2B was to show that ALDH+ cells co-stain with many CGA+ cells in normal crypts, but ALDH+ cells typically do not co-stain with CGA+ cells in CRCs. The other purpose of the data given in Figures 2C-2F was to show our quantification of the crypt location and proportion of cells staining positively for CGA and ALDH1. This study involved a large analysis to score stained cells in hundreds of co-stained crypt sections and was not designed to record images to qualitatively show the distribution of stained cells in individual crypts that was already shown in Figures 1A and 1B. Nonetheless, we now provide representative images of co-stained crypts in the supplemental materials section. We also now show the crypt level for all figures in the main text.

Minor concerns:
1. Layout of the results is difficult to follow. It would be helpful if the results and figures were in the same order.

►We have revised the order of the results to make it easier to follow the figures.

2. In general, the figures are of poor quality. In addition, the IHC and IF images are lacking scale bars and would benefit from arrows to depict areas of interest. The text in most figures needs to be improved, and this is particularly evident in Figure 4E and F, where the green text is illegible. Similarly, the panels in Figure 5D don’t show anything.

►Thank you for this suggestion. We have now added scale bars and arrows pointing to areas of interest in IHC and IF images. The text in the figures has been improved, particularly in Figure 4E and F. We have now increased the contrast and added black background to improve visualization of the phase contrast images in Figure 5D (now Figure 5E) and the quantification of ALDH+ cells represented in Figure 5E panels is given in Figure 5F.

3. It would be helpful to have the crypt levels shown on one example IHC and IF image to help orient the reader.

►Thank you. We have now added crypt levels to IHC and IF images.

4. The y-axes in Figure 2 need to be labeled. As it is now, C-E show the crypt level and then there is a switch to % of the cell population. This is not labeled and left up to reader interpretation.

►The y-axes in Figure 2 are now labeled in our revised manuscript.

5. In the results and figure legend, Figure 3 is said to have multiple specific panels; however, the image only shows A and B (B is never described).

►We apologize and have corrected this error.

**Reviewer #2:**
MAJOR:
1. The studies are largely observational and descriptive in nature. The title indicates a link between RA and WNT signaling, but this is not mechanistically tested in this paper. Instead, the authors cite a number of previously studies to support their conclusions.

►We have toned down our main conclusion and have changed the title.

2. The mechanistic studies focus on GLP-2 and SST signaling, which are well conducted and support the major conclusions of the paper. The title should mention these mechanistic observations.

►The title has been changed to reflect mechanisms involving delay in neuroendocrine cell maturation and altered GLP-2 and SST feedback signaling.

3. Many of the conclusions are based on amalgamation of previously published work. The conclusions should be tempered and based on data presented in the current paper.

►We have toned down our main conclusion to indicate that the data in the current study, in context with our previous published data, provides a clue to a link between RA and WNT signaling in CRC development. This idea was conceived during the current study in considering a mechanism that explains our research findings. Since this mechanism has not been described before, we believe that it is an innovative aspect of our study and worthwhile stating particularly since our results provide a strong clue to the possibility for the mechanism.

4. The model in Figure 4 refers to a feedback mechanism involving the regulation of progenitor SC proliferation by mature NECs. It’s not clear that this model is fully supported by the data presented.

►Our assumption was that cell division is regulated by a feedback mechanism. This assumption was based on the fact that many feedback mechanisms in growth processes in biology involve feedback stemming from the products to the reactants in order to regulate the rate of generation of the product. Thus, we assumed that the mature cells feedback either on stem cells or on progenitor cells. In our iterative modeling to fit the data, the model fit with feedback occurring between mature cells and the progenitor cells not between mature cells and stem cells. Indeed, when we performed the biological experiments we did not find any feedback between mature cells and stem cells only between mature cells and progenitor cells. Thus, the biological data concurred with the modeling result. We have revised the manuscript to clarify the rationale for our assumptions.

MINOR:
1. Page 5: Reference to 1/2 or 1/20 individuals who develop adenomatous polyps or colon cancer, respectively. Do these numbers refer to the general population? Please clarify.

►They refer to the general population. We have clarified this point and including citation of the reference.

2. Page 7: 5-HT is referred to as a neuro-peptide. This is incorrect. It’s a catecholamine.

►We apologize and have corrected this mistake.

3. Figure 1C, D and E: curve axes should have the same scale to enable more meaningful comparison.

►We have now added scale bars to Figures 1C, 1D and 1E to enable more meaningful comparison.

4. Figure 2B: ALDH staining is much weaker in colon cancer specimens compared to normal crypts. What’s the explanation?

►The difference in ALDH staining intensity in colon cancer specimens compared to normal crypts is due to technical variations. We have now added a different image of ALDH staining in normal crypts that is a more appropriate comparison with that of colon cancer.

5. Quantitation of data in Figure 3 should be shown, similar to Figure 2.

►The crypt location of the neuroendocrine cell types in Figure 3 is provided in Table 1. We now mention in the text that the crypt distribution of these other NEC types (noted in Table 1) is similar to that of CGA+ cells (Figure 1C). Because the proportion of these other NEC types is small, we were not able to create scatter plots similar to Figure 2. The main purpose of Figure 3 was to show that staining for other NEC markers co-stains with CGA, and that SSTR1 cells and GLP-2R are different NEC types which each express their own respective neuropeptides SST and GLP.

6. Figure 3 panels are mislabeled as A and B. The text refers to panels A through J.

►We apologize and have corrected this mistake.

7. Raw flow cytometry data should be included for plots in Figure 5. The raw data could be added as supplemental material.

►We have now included this data in supplemental material.
